# MoEKAN: Multi-Scale Transformer-Based Gating KAN Experts Network for Time Series Forecasting

**DOI:** 10.3390/s25237287

**Published:** 2025-11-29

**Authors:** Donghyun Kim, Jimyung Kang, Hoseong Hwang, Hochul Kim

**Affiliations:** 1 Department of Medical Artificial Intelligent, Eul-Ji University, Seongnam-si 13135, Republic of Korea; 2 Korea Electrotechnology Research Institute, Ansan-si 15588, Republic of Korea; 3 Department of Radiological Science, Eul-Ji University, Seongnam-si 13135, Republic of Korea

**Keywords:** OLTC, time series forecasting, Kolmogorov–Arnold networks, mixture-of-experts

## Abstract

An on-load tap changer (OLTC) is a critical component of power transformers, and the vibration signals generated during its operation provide valuable information for forecasting equipment conditions and anomalies. In this study, we propose a novel mixture-of-experts-based Kolmogorov–Arnold network (KAN) model, referred to as MoEKAN, to enhance the accuracy of time series forecasting for the vibration signals of the OLTC. The proposed MoEKAN incorporates reversible instance normalization (RevIN) to flexibly adapt to changes in data distribution and employs a transformer-based gating mechanism to dynamically integrate forecasts from various KAN expert models. In addition, multi-scale signal processing is performed to effectively capture the complex periodicity and patterns present in the vibration data. Experiments using real OLTC operational data demonstrate that the MoEKAN model achieves superior forecasting performance, recording forecasting errors with MSE, MAE, and MAPE values of 133.4579, 7.2801, and 4.4272, respectively, outperforming all comparison models. These results validate the practicality and contribution of the proposed model and confirm its potential as a highly reliable diagnostic tool for condition monitoring and predictive maintenance of OLTCs.

## 1. Introduction

In power systems, transformers are critical equipment, and among their components, the on-load tap changer (OLTC) plays a critical role by adjusting the secondary-side winding taps of the transformer in real time to ensure a stable output voltage. By changing the taps under a load, the OLTC dynamically regulates the output voltage, reducing the voltage deviations and flickering and thereby improving the overall power quality [1]. Maintaining a stable voltage also prevents overvoltage and undervoltage damage to electrical equipment, thereby extending equipment lifespan and enhancing overall system reliability [2]. Therefore, the operational reliability of the OLTCs in large power transformers is extremely important for maintaining power quality and equipment stability. However, despite its importance, the OLTC is a vulnerable component with frequent failures. According to recent studies, more than 20% of transformer failures originate from the OLTC, indicating that OLTCs have a relatively higher failure rate than other parts [3]. Field cases show that transformer failures often stem from issues in major components, such as OLTCs or high-voltage bushings, and such failures directly lead to a shortened transformer lifespan [4]. If an OLTC malfunctions, controlling the output voltage becomes difficult, resulting in voltage instability that can degrade power quality and damage electrical equipment. Therefore, fault diagnosis and condition monitoring of OLTCs are essential for extending the transformer lifespan and ensuring the reliability of power systems.

Conventional OLTC fault diagnosis and maintenance methods exhibit several limitations. Traditionally, the maintenance of transformers and OLTCs relies heavily on time-based maintenance—scheduled inspections at fixed intervals. In this reactive approach, anomalies are often recognized only after a fault has occurred or the damage has already progressed, making immediate remedial action difficult [5]. Moreover, even regular preventive maintenance at fixed intervals cannot completely eliminate the risk of unexpected failures occurring between inspections and can lead to unnecessary equipment downtime [5]. In practice, if defects such as mechanical wear or poor contact in an OLTC accumulate to a critical level, they can result in sudden failure without clear warning, causing equipment shutdown and interruption of the power supply. To overcome these limitations, it is necessary to move beyond simple post-fault diagnostics toward real-time condition monitoring and early anomaly detection while the equipment is in operation.

Recent research has begun to adopt data-driven real-time monitoring and time series forecasting techniques as part of condition-based maintenance (CBM) strategies to forecast OLTC failures. In particular, time series analysis using vibration sensors is considered as an effective approach for the prompt detection of subtle mechanical anomalies or contact wear in OLTCs [6]. Faults in OLTCs often manifest as slight anomalous signs in the vibration and current signals from a few seconds to tens of seconds before the fault fully develops [7]. Thus, even securing a short forecast window on the order of 1 min can yield substantial practical benefits. For example, if an impending OLTC abnormality can be forecasted just a single minute ahead, an operator can immediately halt the tap-changing operation and trigger an emergency OLTC shut-off to protect the equipment and implement protective measures, such as rapidly reducing the transformer load or switching to a bypass [8]. In addition, an immediate warning alert can be sent to operators and maintenance personnel to enable field engineers to quickly perform urgent inspections and initial repairs. Furthermore, operators can instantly adjust the OLTC temperature controls or operating voltage set-points to prevent further damage and even initiate contingency plans, such as swiftly transferring the load to the backup equipment. Therefore, a short-term time series forecast of approximately one minute is critical for immediate fault prevention and enhancing the reliability of OLTCs and transformers.

Various types of data, including acoustic, current, voltage, and vibration data, can be used as inputs to predict transformer and OLTC faults. Vibration data immediately and sensitively reflect changes in the internal mechanical state of the equipment. Voltage and current measurements indicate the electrical flow and state of the system. However, when early fault symptoms or mechanical issues occur, these electrical signals often exhibit only minor changes that are difficult to detect at an early stage and show significant deviations only after an anomaly has progressed to a noticeable level [9]. Furthermore, acoustic data are highly susceptible to external noise and environmental factors, causing them to contain irrelevant signals unrelated to actual equipment anomalies, potentially leading to false alarms [10].

In contrast, vibration data can more accurately and sensitively capture internal mechanical movements, contact conditions, wear and lubrication status, and the tap changer operating state. Any incipient mechanical anomaly or sign of damage inside an OLTC or transformer are reflected immediately in the vibration signals, and clear anomalous indications appear earlier in the vibration data than in other types of signals [11]. Moreover, vibrations can be measured at a relatively high frequency, enabling the precise capture of subtle mechanical changes that occur in the initial stages of a fault. Therefore, vibration data are considered the most suitable source for the fault prediction in OLTC and transformer equipment. In particular, the ability to detect minute anomalous symptoms from a few seconds to tens of seconds before a failure allows for a rapid emergency response, making vibration-based predictive approaches superior to those that rely on acoustic, current, or voltage data.

Early monitoring of the OLTC vibrations relied on simple threshold-based rules to check whether the measured signal exceeded a predefined threshold. In this approach, an alarm is triggered when vibration sensor readings exceed a fixed threshold value. While this method is easy to implement and the criterion is straightforward, real vibration signals contain various noises and long-term drifts; therefore, using a static threshold to detect anomalies often leads to frequent false alarms and may fail to identify subtle or evolving anomalies [12]. For example, if an indicator derived from the OLTC motor current gradually changes owing to environmental factors such as temperature, it becomes difficult to set an appropriate fixed threshold; consequently, threshold monitoring alone may fail to catch a developing vibration anomaly in time [12]. In practice, threshold-based techniques cannot account for equipment-specific characteristics or environmental changes, limiting their ability to provide reliable alerts; furthermore, there are concerns about confusion caused by unnecessary alarms or the possibility of missed alarms [12]. This led to the recognition that simple threshold monitoring is inadequate to meet the requirements of predictive maintenance.

The introduction of statistical time series forecasting methods and early machine learning techniques has marked an important turning point in overcoming these limitations. Instead of fixed thresholds, researchers proposed approaches that forecast the normal pattern of a vibration signal over time and determine anomalies based on the difference between the forecast values and actual measurements. For instance, methods using autoregressive integrated moving average (ARIMA) models or adaptive Kalman filters have been studied to forecast future OLTC vibration values and detect signs of anomalies from forecasting errors. Machine learning-based analyses have also been attempted to handle the complex characteristics of vibration signals. For example, one reported case used a wavelet transform to extract OLTC vibration features and a support vector machine (SVM) optimized by a genetic algorithm to classify the OLTC condition as normal or abnormal [13]. These data-driven approaches allow for a more sophisticated interpretation of vibration patterns than simple threshold rules. Furthermore, some studies have shown that statistical models or shallow machine learning techniques achieve improved anomaly detection performance compared with threshold-based methods. However, these early methods have limitations; their performance is highly dependent on the choice of input features and data quality. In other words, effective forecasting and classification require manual feature extraction guided by domain expertise. If feature engineering is not performed well or if new unforeseen conditions arise, the performance could degrade, indicating a lack of flexibility in these approaches [14].

Against this backdrop, the advent of deep learning techniques has further enhanced OLTC vibration forecasting capabilities. In particular, recurrent neural network (RNN) models that are well-suited for learning time series data have been utilized to model complex temporal patterns in the vibration signals. RNN variants such as long short-term memory (LSTM) networks and gated recurrent units (GRUs) can consider long-range dependencies by retaining information from many past time steps and automatically capturing hidden features that are not predefined by humans. LSTM networks have been widely used in industrial equipment prognostics, and they have demonstrated excellent performance in detecting anomalies in irregular time series data [15]. This suggests that the ability of an LSTM to learn complex patterns even in noisy signals, such as vibration data, can contribute to improved forecasting accuracy [15]. A major advantage of deep learning models is that multi-layer neural networks can learn features directly from the data, significantly reducing the need for complex manual feature extraction that was essential in previous methods, and thereby lowering the dependence on prior expert knowledge [16]. For example, a deep autoencoder-based model can automatically learn important patterns from vibration signals and analyze the reconstruction error, facilitating the detection of subtle anomalies that conventional statistical methods might miss at an early stage [17]. With the adoption of deep learning, it has become possible to learn intricate patterns and future trends from large volumes of complex vibration data, and numerous studies have reported that forecasting accuracy and anomaly detection sensitivity have improved compared to traditional statistical or shallow ML methods.

With advancements in deep learning, the emergence of Transformer-based models has led to another leap in time series vibration forecasting. The Transformer, originally a breakthrough in natural language processing with its self-attention mechanism, has been applied to time series data and has demonstrated its strength in effectively capturing long-range dependencies [18]. Unlike RNNs, the Transformer architecture considers the relationships between all time steps in parallel, which means that even changes from the distant past can influence current forecasts. This characteristic is extremely useful for vibration forecasting problems in which long-term patterns, such as seasonal cycles or trends, are important. Studies applying Transformers to time series forecasting have reported outstanding performance in long-horizon forecasting, a task that is challenging for RNN-based models such as LSTMs [19]. For example, a vehicle vibration prediction study proposed a model that combined a Transformer architecture with recurrent and convolutional networks to achieve highly accurate long-term vibration forecasts [19]. This model applied an attention mechanism to the features extracted from an input sequence, effectively learning long-range patterns and significantly improving the accuracy of vibration prediction over a longer future interval compared to conventional sequential prediction methods [19]. Additionally, Transformers allow parallel computation, which makes them advantageous for handling large datasets, and they can flexibly accommodate variable input and output lengths, making them well-suited for multistep forecasting of multiple time series simultaneously [20].

Very recently, a new neural network concept called the Kolmogorov–Arnold network (KAN) has emerged, offering fresh possibilities for vibration time series forecasting. KAN is based on the Kolmogorov–Arnold representation theorem and uses a continuous function composition approach to improve both the expressive power of the model and its interpretability [21]. While a traditional multi-layer perceptron (MLP) uses fixed activation functions, a KAN assigns each weight a learnable univariate function (for example, a B-spline) so that the network learns the functions themselves. That is, instead of linear weights, smooth spline functions reside in each connection to perform nonlinear transformations on the inputs, with the nodes performing only simple summations. Because of this innovative structure, the KAN can achieve an accuracy equal to or greater than that of a large MLP on the same problem with a much smaller model size [22]. Initial research has shown that as the network size grows, the accuracy of KAN improves according to scaling laws that are more favorable than those of an MLP. This suggests that KANs can mitigate dimensionality limitations and efficiently learn complex functions even for high-dimensional data [22]. Another strength of the KAN is its transparency and interpretability. Using a KAN, a trained network can be analyzed and unnecessary nodes or learned functions can be pruned, thereby simplifying the model into a human-understandable mathematical form [23]. Furthermore, there have been cases in which the pruning of the functional relationships learned by a KAN yielded equations close to physical laws, effectively allowing a neural network to rediscover physical principles on its own [23]. This type of white-box capability is in stark contrast to the black-box nature of conventional deep learning models and can be a major advantage in the predictive maintenance of power equipment, where safety and trust are paramount.

Although KAN is still in the early stages of research, its application in the power domain has already begun. For example, one study successfully used a KAN to diagnose the mechanical condition of a power transformer, demonstrating that it could reliably handle complex field data [24]. Another study reported that combining a KAN with existing prediction models greatly improved the multi-step forecasting performance and stability of a power demand time series [25]. These results suggest that the KAN has high potential as a next-generation forecasting model.

Recent studies have made notable progress in vibration-based machinery analysis. For example, the work in [26] introduces an enhanced time–frequency representation using a frequency–chirp-rate synchrosqueezing-based scaling chirplet transform to better characterize nonstationary vibration components. The study in [27] proposes a domain-adaptive Transformer framework that improves cross-domain fault diagnosis performance under varying operating conditions. Similarly, the method in [28] employs an entropy-guided chirplet transform to adaptively refine instantaneous frequency estimation for complex multi-component vibration signals. In addition, the model in [29] presents a multi-sensor feature fusion approach based on cross-attention, effectively integrating heterogeneous vibration channels for high-accuracy fault classification.

However, despite these advancements, these studies predominantly focus on fault diagnosis, feature extraction, or time–frequency representation enhancement rather than long-horizon vibration forecasting, leaving the problem of predicting future vibration behavior largely unexplored. Moreover, many of these methods depend on high-frequency, multi-channel sensor configurations and computationally intensive preprocessing pipelines, which are not always practical for low-bandwidth or single-sensor monitoring scenarios such as OLTC installations. Finally, none of the above approaches explicitly addresses the highly nonstationary and multi-scale temporal dynamics characteristic of OLTC vibration signals. These limitations highlight the need for a forecasting-oriented framework capable of modeling multi-scale temporal patterns, operating effectively on univariate or low-frequency vibration data, and remaining robust under real-world nonstationary conditions.

**Figure 1:**
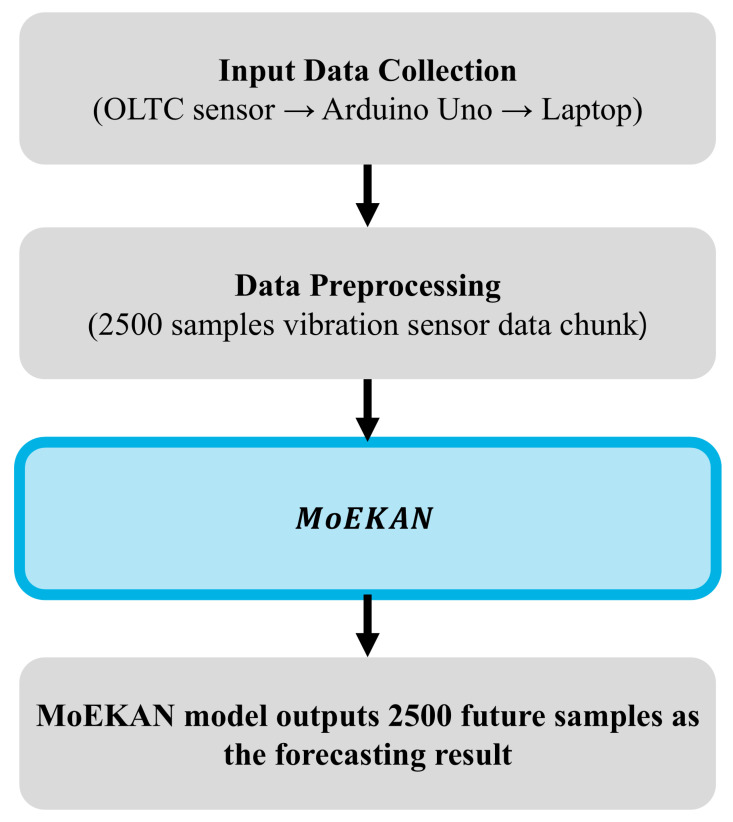
Overview of the proposed MoEKAN-based OLTC vibration forecasting system.

In this study, we propose a model called mixture-of-experts KAN (MoEKAN) to effectively address the OLTC vibration data forecasting problem. MoEKAN is a novel architecture that integrates multiple expert networks based on the KAN theory. By learning optimal nonlinear transformations from data without prefixing any node activation function, the MoEKAN achieves a superior representational capacity compared with existing forecasting models. We also introduce a multihead attention-based gating mechanism that aggregates the features extracted by each expert network in a comprehensive manner, thereby efficiently learning both short- and long-term temporal patterns, as well as global context information from a vibration time series. This structural improvement enables the model to accurately capture even subtle vibration changes during OLTC operation and effectively model complex nonlinear relationships while preserving the temporal structure of the vibration signal. Another major contribution of our study is the direct experimental validation of the performance of the proposed MoEKAN model using OLTC vibration data collected from a real industrial environment. Through various experiments, we demonstrated the practical feasibility of MoEKAN and confirmed that it achieved excellent performance in forecasting the vibration behavior of an operating OLTC. This demonstrates that the proposed approach can effectively contribute to OLTC condition monitoring and early fault diagnosis. The overall structure of the proposed method can be seen in detail in Figure 1.

In summary, the key contributions of this study are as follows:

- Proposal of a MoEKAN-based model: We developed the MoEKAN, a multi-expert architecture leveraging KAN principles. By learning optimal nonlinear transformations directly from data without any pre-specified activation functions, this model significantly enhances the feature representation capability for vibration forecasting compared with prior approaches.
- Multi-head attention gating for global context learning: We designed a gating network using a multihead attention mechanism that dynamically weighs and combines the diverse temporal patterns captured by each expert. This enables the effective learning of long-term dependencies and the global context, integrating multi-scale characteristics of OLTC vibration signals (from local impulse patterns to long-term trends) into the model without loss.
- Experimental validation on real OLTC data: Using a vibration dataset collected from an actual OLTC in the field, we validated the forecasting performance of the MoEKAN and demonstrated its practicality. The results show that our model outperforms the existing methods, proving that MoEKAN is a practical and effective solution for OLTC vibration forecasting and early fault detection.

The remainder of this paper is organized as follows. Section 2 reviews the latest deep-learning-based models for time series forecasting and analyzes their strengths and weaknesses, with a particular focus on recent research related to KAN-based models. Section 3 provides a detailed description of the composition and characteristics of the vibration dataset collected from an actual industrial OLTC system, along with the data processing steps for the forecasting task. Section 4 presents the detailed architecture of the proposed MoEKAN model, including the design and operation of RevIN-based normalization, transformer-based gating mechanism, multi-scale feature handling, various KAN expert network structures, and attention–softmax-based methods for combining expert outputs. Section 5 explains the experimental setup used for model training and evaluation and provides a quantitative performance comparison between MoEKAN and various existing forecasting techniques, along with an analysis of the results. Finally, Section 6 summarizes the key findings and conclusions of the study and discusses future research directions and potential improvements.

## 2. Related Works

Time-series forecasting for condition monitoring and predictive maintenance addresses the challenging problem of predicting future equipment behavior from historical sensor measurements, rather than merely detecting already manifested faults. In this context, deep-learning-based models have been widely adopted because they can automatically learn temporal dependencies from raw vibration, current, and acoustic signals, and numerous studies have reported that they outperform classical statistical approaches on long-horizon forecasting benchmarks [30,31,32,33,34,35,36,37,38,39]. Existing methods for long-sequence forecasting can be broadly grouped into several architectural families: lightweight linear models that map a fixed-length history directly to a multi-step future with low computational cost [30,31]; convolutional neural network (CNN)-based models that exploit local receptive fields and multi-scale temporal patterns [32,33]; Transformer-based architectures that capture global temporal dependencies via self-attention mechanisms [34,35,36,37]; and, more recently, Kolmogorov–Arnold network (KAN)-based models that aim to combine strong expressive power with improved interpretability for complex time-series relationships [38,39]. In the following subsections, we briefly review representative approaches from each of these families and discuss their strengths and limitations in the context of vibration-based OLTC time-series forecasting, which motivates the proposed MoEKAN framework.

### 2.1. Linear-Based Methods

Linear models for time series forecasting have recently gained attention owing to their simplicity in contrast to complex deep-learning architectures. Zeng et al. (2023) [30] reported that a simple approach using a single linear layer, which uses a fixed-length historical sequence as the input and directly forecasts multiple future points, outperforms several Transformer-based deep models on long-term forecasting benchmarks. This suggests that the excessive complexity of deep models can be a drawback for long-range forecasts and that simple linear mapping may better generalize the overall patterns in data. Linear models are extremely simple in structure with very few parameters, leading to fast training and inference. This light weight is a major advantage for applications such as real-time forecasting. However, if used without additional processing, a single linear model is vulnerable to time series non-stationarity (changes in data distribution over time) and may be sensitive to shifts in the distribution of input data. Consequently, recent research has focused on combining linear models with techniques to adjust for trends or scale changes in data to improve performance.

A representative example is the NLinear [30] model, an improved linear approach that introduces a simple preprocessing technique to correct recent trends in a time series. Specifically, NLinear uses the last value of the input sequence as a baseline and subtracts this value from all points in that input window, effectively normalizing the sequence in terms of “change relative to the last time point.” The linear layer only needs to learn the extent to which each future value deviates from the most recent observed value. The output of the linear layer is produced in a delta form (increase or decrease from the last value), and the final forecast is obtained by adding back the last input value (which was previously subtracted) to the output of the linear layer, thus restoring the original scale of the data. This pre-processing allowed the model to focus on the remaining patterns of change by removing the local level (recent trend) from the input sequence. As a result, NLinear achieves forecasting accuracy on various time series datasets that is comparable to, and sometimes better than, those of complex Transformer-based deep learning models. An important advantage of NLinear is that this normalization step is dynamically computed for each data window using a very simple procedure with no additional learned parameters, allowing the model to inherently adjust for a certain degree of nonstationarity in the input before forecasting. This improves the generalization performance of the model by feeding it a detrended input. However, the linear layer itself cannot capture complex nonlinear relationships; it models the contribution of each variable as a weighted sum with fixed weights. Thus, the overall representational power is limited. In essence, linear models, such as NLinear, are constrained to learning only the linear relationships present within an input window.

RLinear [31], proposed by Li et al. (2023) [31], applies the reversible instance normalization (RevIN) technique to a linear forecasting model, providing a more extensive correction for distributional shifts. RLinear operates in the following three steps: First, for each variable in an input window, the RevIN-based normalization subtracts the mean, divides it by the standard deviation of that window (for that variable), and saves these statistical values. This removes the particular level (mean) and scale (variance) of the segment and standardizes the input distribution. Second, the normalized input sequence is passed through a single linear layer to forecast future values in the normalized space. Finally, inverse normalization is applied to the linear output using the previously saved mean and standard deviation, transforming the forecasts back into the original distribution space. Because RevIN normalization is applied consistently during training, the model can learn stable linear mapping even if each training window has very different means or variances. Furthermore, the data in each window are first standardized; thus, the linear layer always sees a relatively stationary distribution. This design enables RLinear to handle a wider range of nonstationarity (e.g., seasonal variance changes or sudden distribution shifts) more robustly than NLinear. Despite its simplicity, RLinear achieved high forecasting accuracy on multiple real-world datasets, often outperforming Transformer-based complex models, demonstrating that even a simple linear model can be used for long-term forecasting when enhanced with proper normalization. In summary, RLinear maintains the lightweight and simple structure of a linear model while using normalization to adapt to data shifts. Furthermore, it demonstrates long-horizon forecasting performance comparable to those of complex deep models.

A comparison of NLinear and RLinear (two linear-series models) highlighted their structural similarities and differences. Both models fundamentally use a single linear transformation to map past time series directly to future values; therefore, they share an extremely simple architecture and the same overall framework. However, they differ in how the input features are processed. NLinear performs simple shift normalization based on the last value of the input window, whereas RLinear applies statistical normalization by adjusting the mean and variance of each window. The methods of NLinear remove only the recent level within the window (correcting a local trend), whereas those of RLinear correct broader distributional changes by standardizing the entire segment. This distinction also affected the representation of temporal dependencies. The linear layer of NLinear is primarily suited for learning short-term increase/decrease patterns relative to the recent level, whereas that of RLinear on standardized inputs and, therefore, can more easily capture consistent long-term patterns, even when the data segments have heterogeneous scales or trends. However, neither model can explicitly capture nonlinear relationships or complex interactions, because both rely on a single linear mapping over the input window; their ability to represent dependencies is limited to the linear correlations present in that window. In terms of the forecasting process, both models output a multivariate multistep forecasting directly from the linear layer without a separate decoder network, and each model then applies its respective inverse normalization to restore the results to the original scale. Specifically, NLinear simply adds the last input value back to each output to recover the original scale, whereas RLinear reapplies the saved mean and standard deviation to the forecasts to return them to the original distribution. Through these restoration steps, both models ensure that their final forecasts align with the scale and baseline of the source data. In conclusion, NLinear and RLinear follow the common paradigm of a “linear model plus normalization,” but they are distinguished by the scope and method of normalization they use, leading to differences in how well they handle various types of non-stationarity in data. In simple terms, both remain structurally limited to linear mappings and thus do not extract nonlinear features such as complex CNNs or Transformers; however, the broader normalization of RLinear gives it greater adaptability to distributional changes than NLinear.

### 2.2. CNN-Based Methods

In time series forecasting, one-dimensional (1D) convolutional neural networks (CNNs) have been widely studied for their computational efficiency and ability to extract local patterns. TimesNet [32] and SCINet [33] are two representative CNN-based models often cited in the recent literature. Each introduces a unique convolutional structure that employs a 2D convolution architecture leveraging periodicity. SCINet uses a hierarchical multiresolution convolution approach, whereas CNNs can segment a time series into shorter subsequences, capture local features in each segment, and allow parallel computations across segments. This makes them well-suited for long time series, as their complexity grows primarily with the convolution kernel size and network depth rather than the sequence length. Unlike linear models, CNNs can learn nonlinear patterns, and compared with Transformers, CNNs do not require computing interactions between all pairs of time points, allowing them to control the computational cost even for long sequences.

TimesNet (temporal 2D-variation network for time series analysis) is a model designed to explicitly exploit multi-periodicity in a time series via a 2D convolutional structure. This transforms a 1D time series into a 2D tensor representation based on the periodic components of the series, often using a Fourier transform to identify dominant periods. For example, if a time series has a prominent period of length T, TimesNet segments the input sequence into chunks of length T and arranges these segments as rows of a matrix. In this 2D representation, the row dimension corresponds to within-period variations (intra-period patterns across one cycle), and the column dimension corresponds to the progression from one period to the next (inter-period trends over successive cycles). This effectively structures temporal patterns on a 2D grid. TimesNet then processes this 2D input using a module called TimesBlock, which is a multi-kernel 2D convolutional block inspired by the inception architecture. TimesBlock is composed of 2D filters of various sizes, enabling the model to simultaneously learn fine-grained patterns within each period and broader trends across periods. In other words, a single TimesBlock operation can capture detailed intraperiod fluctuations and interperiod changes in a unified step, which is more efficient than attempting to extract such patterns directly from a 1D sequence. TimesBlocks can be stacked in layers or applied in parallel over different periods to build a deeper TimesNet architecture. Finally, the 2D feature maps produced by these blocks are consolidated and projected back into a 1D sequence to generate a forecast output. The design principle of TimesNet is to decompose the complex variability of a time series into periodic components, learn each component effectively in a 2D space, and then recombine them, facilitating a single CNN-based framework to capture a variety of temporal patterns. Empirically, TimesNet has consistently demonstrated improved performance over previous models in a wide range of tasks (from short- and long-term forecasting to classification and anomaly detection), validating its effectiveness as a general-purpose time series model. However, the benefits of TimesNet are most pronounced when the data exhibit clear periodicity. If a time series has a weak or no dominant periodic pattern, the advantage gained from the 2D transformation diminishes, and the added 2D processing may become more complex, with limited benefits. In essence, because TimesNet is specifically designed to leverage an explicit periodic structure, its performance improves when the main periods in the data are prominent; however, if the data lack a clear periodic signal, the 2D conversion step might not yield significant gains and can even act as an overhead.

Sample convolution and interaction network (SCINet) follows a different approach. It is a hierarchical CNN architecture that repeatedly downsamples the time series and extracts features at multiple resolutions. Then, it recombines them to make a forecast. The core idea of SCINet is based on the observation that even if we split a time series into a lower-resolution form (e.g., taking every other point), much of the useful temporal structure is preserved. Specifically, a single SCINet layer divides the input sequence into two sub-sequences, one consisting of even-indexed time points and the other consisting of odd-indexed time points. Each of these downsampled sequences is then fed through several 1D convolutional filters to extract features and capture the complementary aspects of the time series in each part. After the convolution step, an interaction stage recombines the two branches, and the extracted feature representations from the two subsequences are merged back together to form a single output sequence for that layer. This completes a single SCINet layer. The combined output can then be passed to another SCINet layer, which splits it into two parts and repeats the convolve-then-interact process. By stacking layers in this manner, SCINet progressively obtains shorter (coarser) representations of the time series at higher levels, enabling it to capture longer-term patterns and more global trends in the upper layers. Finally, after a certain number of layers, the model reverses the process; it takes the high-level combined representation and reconstructs the full-resolution time series in a step-by-step upsampling manner, ultimately yielding a multi-step forecast. For example, in a two-layer SCINet, the original series is first split and processed (conv + merge) in layer 1, and then the result is split and processed in layer 2. Subsequently, the reconstruction begins by merging the outputs from layers 2 and 1 to produce the final forecasts. This unique hierarchical structure allows SCINet to simultaneously utilize information from multiple timescales; it captures short-term patterns in the lower layers and gradually incorporates long-term dependencies in the higher layers. Experiments have shown that SCINet achieves a forecasting accuracy superior to standard single-scale 1D CNNs and some Transformer-based models, demonstrating the power of this multi-resolution convolution approach. The advantages of SCINet include an exponentially expanding receptive field with each additional layer (because each layer effectively doubles the time span covered by subsequent convolutions). Furthermore, convolution operations at each layer can be executed in parallel, thereby preserving the computational efficiency. However, the architecture of SCINet is relatively complex, and choosing hyperparameters, such as the method of splitting or number of layers, can be nontrivial. This added complexity means that although SCINet can achieve high representational power, it may require careful model design and tuning in practice.

Although TimesNet and SCINet are CNN-based time series forecasting models, they handle temporal patterns in fundamentally different ways. Regarding the input transformation, TimesNet remaps the time series into a Fourier-based 2D plane to explicitly isolate periodic patterns before feeding the data into the network, whereas SCINet operates directly on the original time series in the time domain, relying on a deep hierarchical structure (via downsampling) to internally learn patterns at different time scales. In other words, TimesNet leverages prior knowledge of the period length to rearrange the input, whereas SCINet does not perform an explicit pre-transformation of the input data but instead captures the structure through its layered architecture. To represent time dependencies, TimesNet uses 2D convolutions to capture intra- and inter-period variations within one convolutional operation, effectively modeling short-term and long-term (periodic) dependencies simultaneously on a 2D grid. In contrast, SCINet captures long-range dependencies through iterative downsampling. As the resolution becomes coarser at higher layers, each convolution covers a broader time span; hence, long-term patterns are learned gradually across layers. Thus, TimesNet targets specific periodic dependencies in one shot, while SCINet accumulates long-term dependency information step-by-step through multiple scales. For generating forecasts, TimesNet has a relatively direct path; after processing the 2D representation, it projects the learned features back into a 1D sequence to output future values. In contrast, SCINet must undergo a more involved reconstruction process, combining information from high-level coarse representations back down to a fine resolution to produce the final forecast. Consequently, TimesNet’s path from features to forecasts is more straightforward, whereas SCINet’s path is more complex, involving feedback between layers during reconstruction. These structural differences influence the types of problems for which each model excels. TimesNet tends to perform exceptionally well when clear seasonal or periodic patterns dominate the data because it explicitly harnesses these regularities. In contrast, SCINet performs well in scenarios where the time series does not have a single dominant period but instead contains a mix of patterns and scales; its flexible multi-scale approach allows it to adapt to various embedded structures without requiring an explicit periodic signal. In summary, TimesNet and SCINet both utilize convolution to handle long-range dependencies in a time series; however, one does so by reorganizing the data into a 2D periodic structure, whereas the other uses a novel hierarchical downsampling architecture. Unlike Transformer-based approaches that use self-attention to directly capture global dependencies, these CNN models rely on structural convolutional innovations to cover long-term patterns. Furthermore, unlike linear models, CNN-based models can capture nonlinear local patterns through convolution. However, because a basic CNN only processes local receptive fields, capturing distant interactions requires either a deep stacking of layers or special architectures (such as TimesNet’s 2D periodic mapping or SCINet’s multiresolution hierarchy), in contrast to Transformers, which inherently connect distant time points via attention.

### 2.3. Transformer-Based Methods

Transformer-based models can capture global temporal dependencies via a self-attention mechanism, which makes them promising for time series forecasting. However, a major drawback is that as the input sequence length increases, computation and memory costs of self-attention increase quadratically, becoming prohibitively large. To address this issue, various structural improvements to Transformers have been proposed. For example, Informer (Zhou et al., 2021) [34] introduced a probabilistic sparse attention mechanism to reduce complexity, and Pyraformer (Liu et al., 2022) [35] used a pyramidal multi-scale attention structure to learn long-term patterns efficiently. Building on this line of research, recent models have improved the efficiency and performance of Transformers by reconsidering how an input sequence is tokenized for the attention mechanism. Two notable examples are PatchTST [36] and iTransformer [37], which demonstrate different strategies for structuring transformer inputs.

PatchTST (from the paper “A Time Series is Worth 64 Words”) proposes an efficient method to handle very long input sequences with Transformers. The key idea of PatchTST is to segment a continuous time series into fixed-length patches and treat each patch as a single token, instead of using every time point as an individual token. This drastically reduces the number of tokens that a Transformer must process, thereby lowering the computational complexity. For instance, if an original sequence is divided into patches of length 64, the number of tokens becomes roughly one-sixteenth (1/64) of the number of time steps, thereby significantly reducing the attention workload. This patch-wise tokenization allows the Transformer to operate feasibly even for very long inputs because the effective sequence length in the model is much shorter. In addition, PatchTST handles multivariate series by separating tokens by each variable (channel). Rather than mixing multiple variable values into one token, it constructs independent token sequences for each variable. This means the model does not directly model intervariable interactions through self-attention; instead, the time series of each variable is treated as an independent sequence of patch tokens. The Transformer then focuses on learning the long-term temporal patterns for each individual variable without interference. Owing to this patching and channel separation strategy, PatchTST can efficiently process extremely long input windows. In practice, it has demonstrated excellent performance in long-horizon forecasting tasks and has often achieved state-of-the-art results on recent benchmarks. It is a frequently cited model for long-sequence forecasting because of its high accuracy and scalability.

iTransformer adopts a contrasting approach by altering the interpretation of the input dimensions without modifying the Transformer architecture. In a standard Transformer for a multivariate series, tokens are typically formed along the time axis; each token represents all the variables at a specific time step. In contrast, iTransformer swaps the time and variable axes; it considers the entire time series of each variable as one token. In other words, for a multivariate series with M variables, the iTransformer creates M tokens, where the i-th token encodes the entire history of the i-th variable. To achieve this, the iTransformer applies a nonlinear transformation (e.g., a small 1D feed-forward network) to the sequence of each variable to produce a single vector embedding that represents the temporal pattern of the variable. These M embeddings then serve as tokens for the Transformer’s self-attention, which is applied along the variable dimension rather than along the time dimension. This approach allows the model to effectively learn complex intervariable dependencies through self-attention across variable tokens. By flipping the input perspective in this manner, the iTransformer addresses a key limitation of the conventional method: in standard time-step tokenization, each token bundles together multiple variables, which dilutes the distinct characteristics of each variable and makes the attention patterns difficult to interpret. In the iTransformer scheme, each token corresponds to a single variable, preserving the integrity of the information of each variable and making attention interactions purely about the relationships between different variables. Structurally, the iTransformer uses the same Transformer components (multihead attention, feed-forward networks, normalization layers, etc.) without any custom modification; the only change is in how the input is represented. Because the number of tokens is now equal to the number of variables (typically much smaller than the number of time steps), the iTransformer can comfortably handle significantly long time series; the self-attention cost grows with the number of variables rather than the sequence length. In experiments with datasets having tens to hundreds of variables, the iTransformer has been able to use long input windows without compromising training feasibility, and it has achieved accuracy on par with state-of-the-art methods in multivariate forecasting. The trade-off of this method is that it completely forgoes the modeling of time-step interactions via self-attention. The iTransformer does not directly connect distant time points through attention; instead, capturing each variable’s temporal patterns is left to a token-embedding stage (per-variable nonlinear transformation). This per-variable encoding is a more restricted way to model temporal relationships than full time-axis attention. However, by minimizing direct cross-variable mixing, it stabilizes training and focuses the model on inter-variable information exchange.

PatchTST and iTransformer are contrasting strategies for enhancing Transformer-based forecasting. Regarding input handling, PatchTST splits the time series along the time axis into patch tokens and separates each variable when fed into the Transformer, whereas the iTransformer essentially swaps axes and creates tokens along the variable axis, treating the entire sequence of each variable as a single token. PatchTST concentrates on capturing long-range temporal dependencies within each variable by enabling self-attention to directly link distant time segments while deliberately not addressing variable-to-variable interactions in attention. However, iTransformer focuses on cross-variable dependencies by employing self-attention solely among variables while relying on a simpler per-variable model to handle temporal patterns. From a feature representation standpoint, PatchTST avoids mixing variables in the input tokens so that the model can devote its capacity to learning the intricate long-term pattern of each time series accurately, whereas the iTransformer condenses each variable sequence into an embedding and emphasizes learning the relationships between variables to improve the overall multivariate forecasting accuracy and efficiency. In terms of output generation, both models perform direct multi-step forecasting using only a Transformer encoder (no separate decoder stage). PatchTST uses the Transformer’s output for each channel to forecast that channel’s future values often by attaching a small forecast head to each channel’s output representation. However, iTransformer takes M variable tokens after the Transformer and projects each token to the future values of the corresponding variable. Thus, the manner in which the final outputs are mapped from the Transformer differs between the two models; however, in both cases, forecasting is performed in one pass. Owing to these differences, PatchTST is mainly oriented towards efficiently handling significantly long time series by reducing the sequence length, whereas iTransformer is oriented towards effectively handling many variables by focusing on intervariable dynamics. Each addresses different challenges and can be viewed as complementary improvements to the Transformer frameworks. These models illustrate that by simply rethinking the formulation of the input sequences, one can significantly improve the efficiency and accuracy of a Transformer in time series forecasting. Compared with the CNN or KAN approaches, PatchTST and iTransformer still rely on deep architectures and data-driven attention mechanisms; however, they demonstrate that creative input structuring can mitigate some of the drawbacks of Transformers. The strength of Transformer-based models lies in the flexibility of self-attention to capture complex and long-range correlations, and with appropriate structural enhancements, they can learn significantly intricate temporal patterns. However, unlike linear or KAN-based approaches, Transformers (even improved ones) tend to have lower interpretability and theoretical simplicity, which is a trade-off to consider when choosing a forecasting model.

### 2.4. KAN-Based Methods

KANs are neural networks based on the Kolmogorov–Arnold representation theorem (KART) [38], which states that any multivariate continuous function can be represented as a composition of a finite number of 1D functions. By leveraging this principle, KANs structure their models to represent complex functions by using combinations of simpler univariate functions. In theory, the KAN approach has higher expressive power and better interpretability than conventional multi-layer perceptrons (MLP) of the same size. In particular, the function decomposition used in KAN can directly incorporate properties such as periodicity or trends into the model architecture, which is advantageous for capturing temporal patterns in a time series. Owing to this characteristic, KANs can often approximate time series data well, even with a shallow network, and the learned components of the function can be examined in mathematical form, providing insights into the model’s decision mechanism. In practice, KAN-based models have demonstrated that despite their simpler structure, they can effectively learn long-period patterns in data, serving as a middle ground between the simplicity of linear models and rich expressiveness of deep learning models.

A recent model in the KAN family, namely reversible mixture of KAN experts (RMoK) [39], was designed to maximize the forecasting performance with only a single KAN layer while preserving the interpretability of the model. RMoK first employs a RevIN step to mitigate nonstationarity in the input time series. Specifically, for each variable in the input window, RMoK normalizes the data by subtracting the mean and dividing by the standard deviation of that window; it, then, records these statistics. After this normalization, core forecasting is performed using a structure called a mixture of KAN experts (MoK) layer. The MoK layer consists of multiple KAN-based expert networks in parallel, along with a gating network that determines how to weigh the output of each expert based on the characteristics of the input. Each KAN expert can use different basis functions or forms, allowing them to specialize in modeling certain types of patterns or distributions in data. The gating network takes the input (or its representation) and assigns a weight to each expert output, effectively selecting and blending the most suitable experts in a given input window. The outputs of the experts are combined (weighted sum) to produce a normalized forecast. Finally, RMoK applies the stored mean and standard deviation in an inverse normalization step, converting the forecast to the original scale and distribution of the data. Despite having only this single RevIN + MoK layered structure, RMoK achieves state-of-the-art forecasting accuracy on a variety of benchmark datasets, demonstrating its practical effectiveness.

Compared to a conventional single-KAN model, RMoK shows clear differences in input processing and pattern representation. A standard KAN uniformly applies the same form of basis decomposition to all input variables, whereas RMoK assigns potentially different KAN experts to different variables or patterns, thereby enabling a more specialized treatment for each component of a multivariate time series. This means that RMoK can more flexibly handle differences in distribution and nonstationarity across variables, as each expert KAN can be tuned to a particular subset of data behavior. Moreover, the gating mechanism can adjust which experts dominate for a given input. In terms of representing temporal dependencies, RMoK (similar to KAN in general) describes the time series relationship as a functional mapping, rather than a sequential state- or attention-based interaction. However, by employing a mixture of multiple KAN experts, RMoK captures complex piecewise relationships in the time series, and each expert captures a certain aspect of the pattern (for example, one expert might capture a seasonal pattern, whereas another captures a shorter cycle or anomaly); subsequently, their combination yields a rich overall representation. Regarding the forecasting procedure, RMoK performs a one-step forecast. After the initial RevIN normalization, it passes the data through the MoK layer and immediately produces a forecast, which is then inverted back to the original scale. There are no deep stacks of layers or iterative decoding. The model essentially maps the normalized input to the output in a single stage, similar to a linear model (albeit with a more complex internal mapping owing to experts). This makes the architecture remarkably simple and efficient in structure—closer to the simplicity of a linear model—even though it achieves high expressiveness through a mixture-of-expert design. In summary, RMoK leverages the theoretical strengths of KAN by using a mixture of experts and reversible normalization to tailor the model to the characteristics of each variable and then integrates them for forecasting, thereby enhancing accuracy and stability. It retains a shallow architecture while achieving a level of representational power and explainability that is competitive with deeper models. Importantly, each component in RMoK corresponds to an explicit mathematical function (owing to the KAN formulation), which gives it a degree of interpretability that is distinct from other deep learning approaches. By using RevIN to adjust for input distribution shifts and a mixture of experts (MoE) to combine specialized learners, RMoK effectively combines the simplicity of a linear model with the flexibility of a deep model.

## 3. Materials

The experiment was conducted using a three-phase OLTC. Specifically, an ECOTAP VPD model (Maschinenfabrik Reinhausen GmbH, Regensburg, Germany) with a rated voltage of 36 kV and a current of 30 A was used. This OLTC employed a vacuum-switching mechanism and had nine tap positions in its base configuration (expandable to 17 tap positions). An LP302-1D loop-powered accelerometer (Connection Technology Center, Inc., Victor, NY, USA) was used for vibration measurements. This sensor has a measurement range of 0–10 g and produces a proportional analog current output of 4–20 mA (4 mA corresponds to 0 g, and 20 mA corresponds to 10 g). The accelerometer was securely attached to the top metal housing of the OLTC via a mounting stud to effectively capture the vibrations generated during tap-change operations.

**Figure 2:**
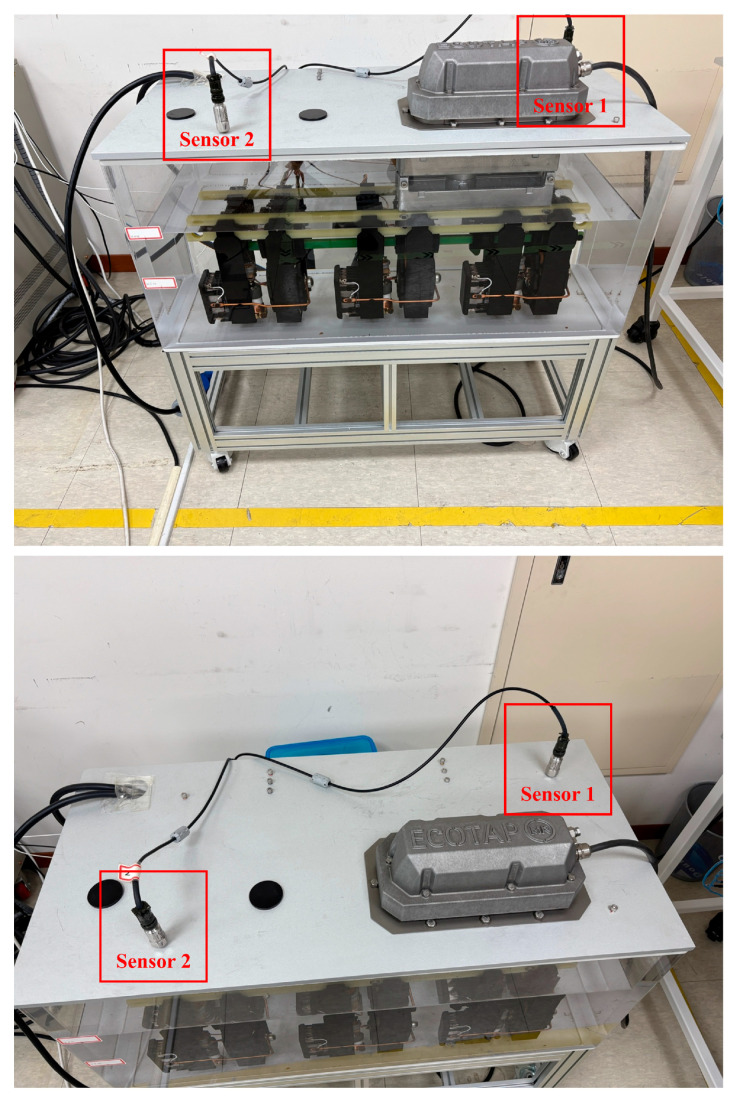
OLTC experimental environment.

To minimize external influences, the OLTC was installed inside a protective acrylic chamber, which served to isolate the apparatus from ambient vibrations and electromagnetic interference (EMI). This isolated environment suppressed extraneous vibrations and EMI from the surroundings, thereby improving the purity of the measured signal. Under these conditions, the vibration signals were recorded during controlled automatic tap-change operations (with tap changes initiated by a controller located outside the chamber). An overview of the OLTC experimental environment is shown in Figure 2.

The analog output of the sensor (current signal of 4–20 mA) was transmitted through a cable to an Arduino Uno microcontroller board (Arduino, Turin, Italy) positioned outside the chamber. Arduino Uno’s built-in 10-bit analog-to-digital converter (ADC) digitized the incoming analog signal. Subsequently, the digital data were sent in real time to a laptop via USB serial communication. A custom software application developed in-house was run on a laptop to visualize the vibration signal in real time and simultaneously log the data into a text file (.txt). The data collected in this manner were immediately available for analysis and later used as the input for a time series analysis aimed at forecasting the vibration signal for one minute into the future.

The vibration data were sampled at approximately 41.7 Hz, yielding roughly 2500 samples per min. This sampling frequency was intentionally selected as a trade-off between temporal resolution and sequence length for one-minute-ahead forecasting: using a much higher sampling rate would produce tens of thousands of samples per minute, which substantially increases the input/output sequence length and leads to a marked degradation of forecasting performance and computational efficiency in our setting. By limiting the sampling rate to 41.7 Hz, we obtain an input and target sequence of about 2500 samples while keeping the sequence length manageable for the proposed MoEKAN architecture. The experimental apparatus and data acquisition configuration were thus designed to provide a robust and sufficient dataset for training a forecasting model to forecast the vibration behavior of the OLTC one minute later.

In total, the experiment yielded a dataset of 780,000 time series data points representing actual vibration signals from the OLTC automatic tap-change operations. This dataset was partitioned into a training set of 380,000 samples, a validation set of 200,000 samples, and an independent test set of 200,000 samples for the objective performance evaluation of the forecasting model. The detailed information of the vibration dataset used in this study is summarized in Table 1. Each data sample (row) consisted of a simple sequential index (Num) and the corresponding 10-bit vibration signal value (ranging from 1 to 1025) obtained from the Arduino ADC; no separate timestamp was recorded for each sample, as the sequential index inherently denotes the temporal order. The dataset was stored as raw data in a text file immediately after acquisition without any preprocessing or filtering, and this raw dataset was directly used for model training and evaluation.

Consequently, the dataset used in this study corresponds to a univariate vibration time series with a single measurement channel. Although the proposed MoEKAN architecture is designed to handle general multivariate time-series inputs (e.g., multiple vibration sensors or additional monitoring variables), all experiments in this paper are conducted on this univariate vibration sequence.

For forecasting modeling, a deep learning approach based on KAN was employed. The model was trained using historical vibration time series data to forecast the vibration level one minute in the future (corresponding to 2500 time steps ahead). All the data shared the same format, and higher vibration values in the dataset corresponded to the larger vibration amplitudes experienced by the OLTC during the tap change process.

## 4. Methods

### 4.1. Base Model: RMoK

The RMoK model adopts a mixture-of-experts architecture based on the KAN to effectively learn temporal features. To efficiently handle distribution shifts in the time series input data, the RMoK model first applies RevIN. RevIN normalization is performed through an affine transformation using the mean ($\mu_{i}$) and standard deviation ($\sigma_{i}$) of each input variable, as described by the following equation:

(1) $${\tilde{x}}_{i,t}=\gamma_{i}\left(\frac{x_{i,t}-\mu_{i}}{\sigma_{i}}\right)+\beta_{i}$$

Here, $x_{i,t}$ represents the input value of variable i at time step t, and $\gamma_{i}$ and $\beta_{i}$ are learnable parameters that adjust the normalization level for each variable.

**Figure 3:**
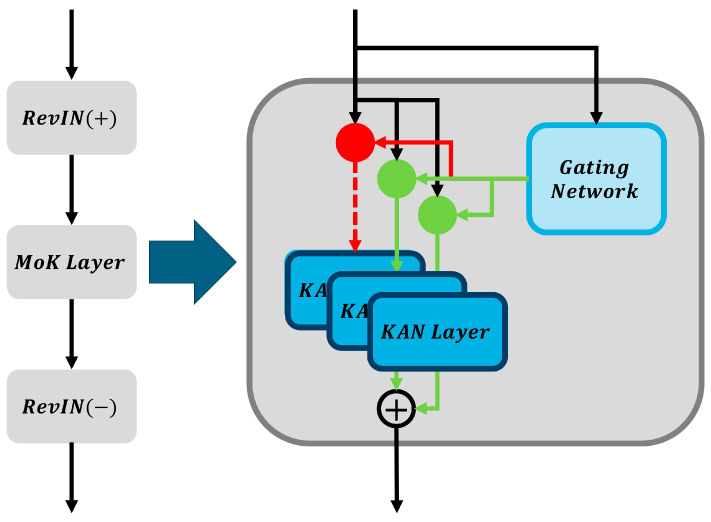
The structure of RMoK and MoK layer.

The normalized input data are then passed to a MoK layer. The MoK layer employs multiple KAN experts of different types to learn diverse characteristics and dynamically determines output weight of each expert via a gating network. The overall structure of the RMoK and MoK layer is illustrated in Figure 3. Each KAN expert, inspired by the KART, transforms the input variables individually through univariate functions $\emptyset_{j}$ and sums these transformed values to produce the output. This process is formulated as follows:

(2) $$y=\sum_{j=1}^{N}\emptyset_{j}(x_{j})$$

Here, $\emptyset_{j}$ represents a learnable univariate activation function, such as a B-spline, which enables the KAN experts to effectively learn nonlinear and complex patterns.

The gating network within the MoK layer generates weights ($g_{i}$) that indicate the relative importance of each expert based on the input data features. A gating network can be implemented in two ways. The first is the softmax gating approach, expressed by the following equation:

(3) $$g_{i}=\frac{e^{h_{i}}}{\sum_{j=1}^{E}e^{h_{j}}},\;\;\;h_{j}=xW_{g,j}$$

Here, $W_{g,j}$ is a learnable weight matrix. The second method is the sparse gating approach, in which Gaussian noise is added to the input to activate only the top k experts, while deactivating the remaining experts. This method is represented by the following equation:

$$h=xW_{g}+Norm\left(Softplus\left(xW_{noise}\right)\right)$$

(4) $$g_{i}^{sparse}=\frac{e^{h_{i}}}{\sum_{j\in TopK(h,k)}e^{h_{j}}},\;\;\;i\in TopK(h,k)$$

Therefore, the MoK layer selects the most suitable experts based on the input data characteristics to perform the forecasting. The outputs from each expert in the MoK layer are combined through gating weights to produce the final forecast, expressed as follows:

(5) $$\hat{y}=\sum_{i=1}^{E}g_{i}y_{i}$$

Finally, the forecast results are restored to the original data scale using the inverse normalization of RevIN. This step utilizes the same statistics and parameters employed during the normalization process and is performed using the following equation:

(6) $${\hat{y}}_{i,t}^{\left(original\right)}=\frac{{\hat{y}}_{i,t}^{\left(norm\right)}-\beta_{i}}{\gamma_{i}}\sigma_{i}+\mu_{i}$$

Here, ${\hat{y}}_{i,t}^{\left(norm\right)}$ denotes the normalized forecast value, and the equation describes the process of reverting it to the original scale.

Meanwhile, when training the RMoK model, a load balancing loss is introduced to balance the weights of the experts, preventing a “winner-takes-all” phenomenon in which the gating network excessively concentrates on a few experts. The load of each expert is measured based on the frequency of its utilization in the forecasts, and the load-balancing loss is defined as the square of the coefficient of variation (CV) of these loads, expressed as follows:

(7) $$L_{load-balancing}={\left(\frac{std(loads)}{mean(loads)}\right)}^{2}$$

Finally, the overall loss function for model training is expressed as the sum of the forecasting and load-balancing losses as follows:

(8) $$L_{total}=L_{pred}+w_{l}L_{load-balancing}$$

Here, $L_{pred}$ is the forecasting loss between the forecast and actual values, and $w_{l}$ is a hyperparameter controlling the importance of the load-balancing loss. This approach guides the model toward achieving accurate forecasts while evenly utilizing all experts.

### 4.2. Proposed Model: MoEKAN

The MoEKAN model employs a mixture-of-experts architecture, in which input time series data are first normalized using RevIN and then combined as a weighted average of forecasts from multiple KAN-based expert models through a gating network to produce the final output. In the input stage, the model is formulated to accept multivariate time-series data that are segmented into windows or combined with additional features as necessary, particularly when employing a multi-scale input processing approach to represent data across various time scales. This design enables the extraction of diverse temporal patterns from long-term trends to short-term fluctuations for each variable, thereby providing rich and informative input features to the model. In this paper, however, the model is instantiated on a single-channel vibration signal (i.e., a univariate time series), and we retain the multivariate notation primarily to highlight that the same architecture can be directly extended to multi-sensor settings.

To further enhance the representation capability, the input sequence is divided into overlapping windows with different lengths, and multi-scale features are extracted from each segment. Short windows emphasize rapid fluctuations and transient behaviors, medium windows capture intermediate dynamics, and long windows reflect slow-varying trends. All windowed representations are temporally aligned with the forecast horizon using causal segmentation to avoid information leakage. The resulting multi-scale features are concatenated along the channel dimension and augmented with scale embeddings that explicitly indicate the temporal granularity. This design enables the model to simultaneously exploit both short-term variations and long-term dependencies, thereby providing a comprehensive description of the vibration signals for subsequent expert selection and forecasting.

In our multi-scale processing module, the original 2500-point input window—corresponding to one minute of vibration data—is used as the long-scale representation. To obtain additional temporal resolutions, we apply simple average-based downsampling. The medium-scale sequence (1250 points) is generated through a 2-to-1 averaging operation, in which every two consecutive samples from the 2500-point window are averaged to form a single value. Likewise, the short-scale sequence (625 points) is produced by applying a 4-to-1 averaging scheme, where every four consecutive samples are averaged. This hierarchical downsampling procedure preserves the essential temporal characteristics across coarse, intermediate, and fine scales while reducing redundancy, enabling the model to capture long-term trends (2500 samples), mid-range variations (1250 samples), and short-term fluctuations (625 samples) within the OLTC vibration signal.

Data distributions are normalized for each variable using the RevIN technique. Each variable $x_{i,t}$ (the input value of variable i at time step t) is standardized using its mean $\mu_{i}$ and standard deviation $\sigma_{i}$, and the scale and shift are adjusted by learnable parameters $\gamma_{i}$ and $\beta_{i}$. This process mitigates time-dependent distribution shifts (nonstationarity) in time series data. RevIN normalization is expressed as follows:

(9) $${\tilde{x}}_{i,t}=\gamma_{i}\left(\frac{x_{i,t}-\mu_{i}}{\sigma_{i}}\right)+\beta_{i}$$

Here, $\gamma_{i}$ and $\beta_{i}$ are learnable parameters that control the intensity of normalization for each variable. After applying RevIN normalization, the model achieves stable learning. Following the forecasting step, inverse normalization is performed using the stored statistical values to restore the results to the original data scale. Inverse RevIN normalization is expressed as follows:

(10) $${\hat{y}}_{i,t}^{\left(original\right)}=\frac{{\hat{y}}_{i,t}^{\left(norm\right)}-\beta_{i}}{\gamma_{i}}\sigma_{i}+\mu_{i}$$

Here, ${\hat{y}}_{i,t}^{\left(norm\right)}$ is the forecast value in the normalized space, and the final forecast ${\hat{y}}_{i,t}^{\left(original\right)}$ is the value obtained on transformation back to the original data scale.

After normalization, the input data, together with multi-scale features, are passed to an expert-level transformer-based gating network. The gating network, implemented as a compact Transformer structure equipped with a self-attention mechanism, learns complex temporal patterns from the time series and extracts representations from the sequence of each variable to calculate the weighting of the most suitable expert for each variable.

Beyond expert-level gating within each scale, MoEKAN employs a scale-level transformer-based gating to fuse forecasts across temporal resolutions. The module applies self-attention across scales, dynamically estimating the relative importance of long-, medium-, and short-term forecasts. The fused output integrates global trends and local fluctuations, ensuring that the final forecast reflects both coarse-grained context and fine-grained variations and improving robustness in long-horizon forecasting.

In contrast to the simple gating mechanism in RMoK, the transformer-based gating in MoEKAN offers four key advantages. (i) With self-attention, it models long-range temporal dependencies, assigning expert weights using both local context and distant history. (ii) It integrates context across variables and time simultaneously, improving expert selection under complex, nonstationary conditions. (iii) Multi-head attention enhances robustness by attending to diverse temporal aspects in parallel, mitigating bias toward a single feature or short-term fluctuation. (iv) The gate adapts dynamically to input variations, maintaining effectiveness under distribution shifts common in real-world vibration signals. These properties make the transformer-based gating more flexible and reliable than a static or linear gating design and significantly enhance the overall forecasting performance of the MoEKAN framework.

In the transformer-based gating network, the gate score $h_{e}$ for expert e is calculated by multiplying the input feature vector x by the gating weight matrix $W_{g}$. This score is normalized using a softmax function to produce the gating weight $g_{e}$, expressed as follows:

(11) $$g_{e}=\frac{e^{h_{e}}}{\sum_{j=1}^{E}e^{h_{j}}},\;\;\;h_{e}=xW_{g,e}$$

This allows the output of each expert to be dynamically combined according to the input. In particular, the transformer-based gating enables the selection of experts based on richer contextual information (e.g., seasonal cycles or trend variations) compared to a simple linear gate, assigning higher importance to forecasts from the most suitable expert at each time step.

The MoEKAN model comprises multiple parallel KAN expert models, each of which independently generates forecasts using the same input data. The KAN layer is a neural network that learns univariate continuous function transformations for each input variable to compute its output. It is mathematically defined as follows:

(12) $$y=\sum_{i=1}^{N}\emptyset_{i}(x_{i})$$

Here, the activation function $\emptyset_{i}$ corresponding to the input variable $x_{i}$ is expressed as a linear combination of basis functions. The proposed model incorporates various KAN experts such as TaylorKAN, WaveletKAN, JacobiKAN, and FourierKAN.

(13) $$\emptyset_{i}\left(x_{i}\right)={TaylorKAN\emptyset }_{i}\left(x_{i}\right)+{WaveletKAN\emptyset }_{i}\left(x_{i}\right)\;\;\;\;+{JacobiKAN\emptyset }_{i}\left(x_{i}\right)+{FourierKAN\emptyset }_{i}\left(x_{i}\right)$$

For example, the activation function of a TaylorKAN expert is represented in a polynomial form as follows:

(14) $${TaylorKAN\emptyset }_{i}\left(x_{i}\right)=\sum_{n=0}^{N}{a_{n}(x_{i}-c)}^{n}$$

The WaveletKAN expert is represented using wavelet basis functions as follows:

(15) $${WaveletKAN\emptyset }_{i}\left(x_{i}\right)=\sum_{m}d_{m}\psi \left(\frac{x_{i}-b_{m}}{a_{m}}\right)$$

The activation function of the JacobiKAN expert is expressed based on Jacobi polynomials as follows:

(16) $${JacobiKAN\emptyset }_{i}\left(x_{i}\right)=\sum_{n=0}^{N}c_{n}P_{n}^{\left(\alpha ,\beta \right)}\left(x_{i}\right)$$

The FourierKAN expert is represented by the following Fourier series to capture periodic characteristics:

(17) $${FourierKAN\emptyset }_{i}\left(x_{i}\right)=a_{0}+\sum_{k=1}^{K}\left[a_{k}\cos\left(w_{k}x_{i}\right)+b_{k}sin(w_{k}x_{i})\right]$$

By arranging experts with distinct characteristics in parallel, the model can effectively learn the diverse patterns present in the input data.

The final forecast was obtained by taking the weighted average of the outputs from each KAN expert using the gating weights produced by the gating network. The final forecast output $\hat{y}$ of the model is expressed as follows:

(18) $$\hat{y}=\sum_{e=1}^{E}g_{e}y_{e}$$

Here, $y_{e}$ denotes the output of the KAN expert e, and $g_{e}$ is the corresponding gating weight obtained from the transformer-based gating network. By effectively combining the outputs of the experts, the model produces optimal forecasts based on the characteristics of the input data.

A load-balancing loss is introduced to prevent the model from focusing exclusively on a small subset of experts. The load of each expert is defined as the proportion of the entire input dataset selected by the expert. Based on this, the loss function aimed at balancing the load among experts is defined as follows:

(19) $$L_{balance}={\left(\frac{std(loads)}{mean(loads)}\right)}^{2}$$

The final loss function is expressed as the sum of the forecasting and load-balancing losses.

(20) $$L_{total}=L_{pred}+w_{l}L_{balance}$$

Here, $L_{pred}$ is the forecasting loss between the forecast and actual values (e.g., mean squared error (MSE)), and $w_{l}$ is a hyperparameter that controls the importance of the load-balancing loss.

Finally, the model forecasts are restored to the original data scale through RevIN inverse normalization (denormalization). This step utilizes the mean $\mu_{i}$, standard deviation $\sigma_{i}$, and learned parameters $\gamma_{i}$ and $\beta_{i}$, which were recorded during the RevIN normalization, and is performed as follows:

(21) $${\hat{y}}_{i,t}^{\left(original\right)}=\frac{{\hat{y}}_{i,t}^{\left(norm\right)}-\beta_{i}}{\gamma_{i}}\sigma_{i}+\mu_{i}$$

**Figure 4:**
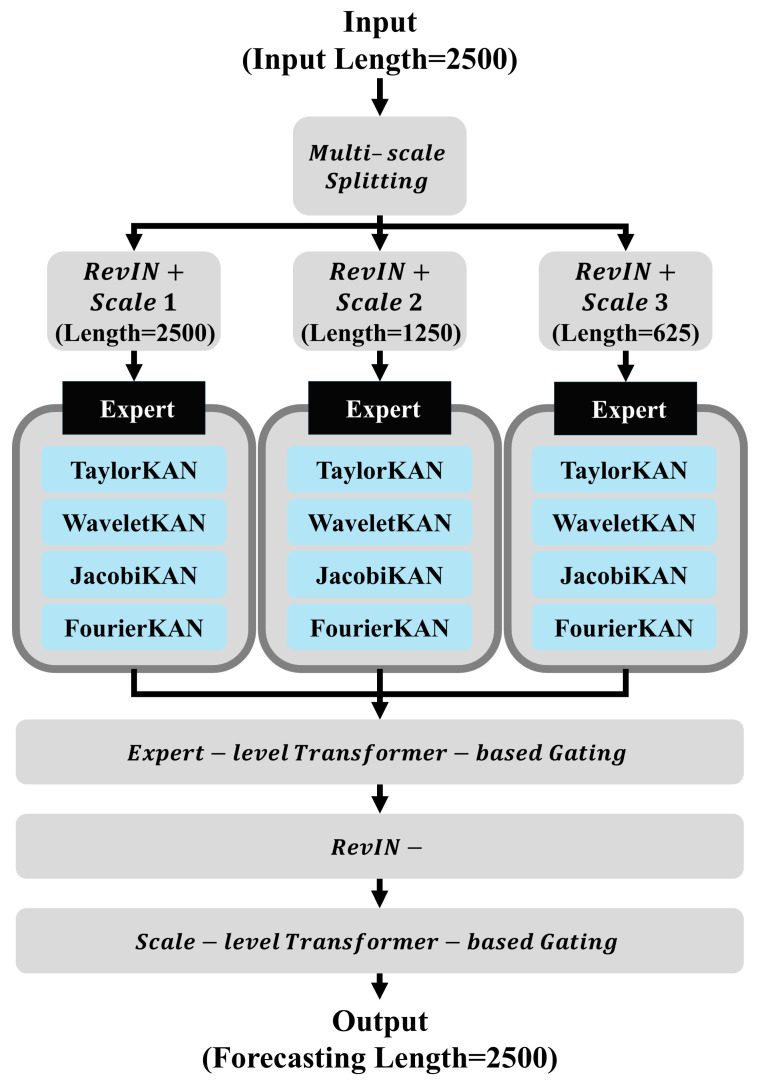
The structure of MoEKAN.

Using this architecture, the MoEKAN model effectively captures the diverse temporal patterns present in the input multivariate time series data, thereby providing stable and accurate forecasts, as illustrated in Figure 4.

## 5. Results and Discussion

Model performance was evaluated using representative time series forecasting metrics, such as mean squared error (MSE), mean absolute error (MAE), and mean absolute percentage error (MAPE). MSE is defined as the mean of the squared differences between the forecast and actual values. It sensitively reflects the magnitude of forecasting errors, yielding relatively large values in the presence of outliers or large errors. MAE is calculated as the mean of the absolute differences between the forecast and actual values, which directly indicates the absolute size of the errors. Because it is less sensitive to large errors than MSE, it is regarded as a more robust metric. Finally, MAPE is defined as the mean of the absolute errors divided by the actual values and expressed as a percentage. By representing the forecasting error as a relative proportion, the MAPE allows for an easy comparison of the model forecasting accuracy across different data scales. In this study, we comprehensively considered these metrics to evaluate the forecasting performance of each model in an objective and balanced manner. The overall performance of all models according to these three evaluation metrics is summarized in Table 2.

Among the linear models, the NLinear model exhibited an MSE of 2127.173, MAE of 28.9779, and MAPE of 20.2196 (forecasting errors). These errors are slightly lower than those of the similarly linear RLinear model (MSE: 2209.712, MAE: 30.3753, and MAPE: 21.4198), indicating that NLinear performs marginally better than RLinear.

RLinear incorporates a technique for normalizing the input data via RevIN prior to forecasting; however, in our experiments, the application of RevIN did not produce a meaningful improvement over NLinear. Overall, both linear models showed a relatively large MAPE of around 20%, suggesting that linear approaches have limitations in capturing the nonlinear complexity of OLTC vibration signals.

For CNN-based models, there was a considerable performance gap between TimesNet and SCINet. TimesNet recorded an MSE of 2939.033, MAE of 39.3729, and MAPE of 19.9801, which were significantly lower than those of SCINet (MSE: 5766.999, MAE: 47.1392, and MAPE: 23.8277). Furthermore, the MSE of TimesNet was only approximately half that of SCINet, and its MAPE (approximately 19.98%) was improved compared with that of SCINet (23.83%). This suggests that the architecture of TimesNet, which transforms a time series into a 2D tensor according to periodicity for learning, effectively captures periodic patterns in the OLTC vibration data, thereby enhancing forecasting accuracy. However, the MAPE of TimesNet remained at approximately 20%, similar to that of the linear models, but its MSE was higher than those of the linear models, indicating that some forecasts had large errors. SCINet exhibited the largest errors in all metrics, indicating that the complexity of the model is ill-suited to the characteristics of the OLTC vibrations, potentially causing overfitting or difficulty in learning important patterns.

The Transformer-based models exhibited a distinct performance improvement over the linear and CNN models. The PatchTST model achieved an MSE of 1122.035, MAE of 18.1859, and MAPE of 11.5839, making it the best performer among the Transformer-based approaches. Compared to the similar Transformer-based iTransformer model (MSE: 1542.971, MAE: 27.3423, and MAPE: 15.8059), the MSE of PatchTST is approximately 27% lower, indicating that PatchTST makes significantly more accurate forecasts than iTransformer. The strong performance of PatchTST can be attributed to its structural advantage of segmenting time series input into patches and learning in a channel-independent manner, which allows it to effectively capture long-term patterns in complex OLTC vibration data. In contrast, iTransformer is a state-of-the-art model that transforms the input dimensions of a Transformer to learn the interactions among variables; however, in our experiments, it did not achieve the same level of accuracy as PatchTST. Nonetheless, the MAPE values of both Transformer models (~11.58% for PatchTST and ~15.81% for iTransformer) were significantly lower than those of the linear or CNN models (>20%), demonstrating the superiority of Transformer-based approaches in learning complex time series trends.

Among the KAN-based models, RMoK exhibited the most notable performance improvement. RMoK, a KAN-based mixture-of-experts model with RevIN, achieved an MSE of 907.3188, MAE of 15.5203, and MAPE of 9.3313. These results represent approximately a 19% lower MSE and a drop in MAPE to single digits compared to PatchTST (MSE: 1122.035, and MAPE: 11.5839), which is the top-performing Transformer model. By using a single-layer mixture structure composed of multiple KAN expert models along with RevIN normalization, RMoK learned the complex patterns of the OLTC vibration data more precisely than the previous models. The substantial decrease in the MAPE value of RMoK to 9.33% suggests that the mathematical expressiveness and interpretability advantages of KAN-based models also contribute to the improved time series forecasting performance. In short, RMoK outperformed all the earlier models, including the linear, CNN, and Transformer approaches, demonstrating the effectiveness of the KAN-based approach.

**Figure 5:**
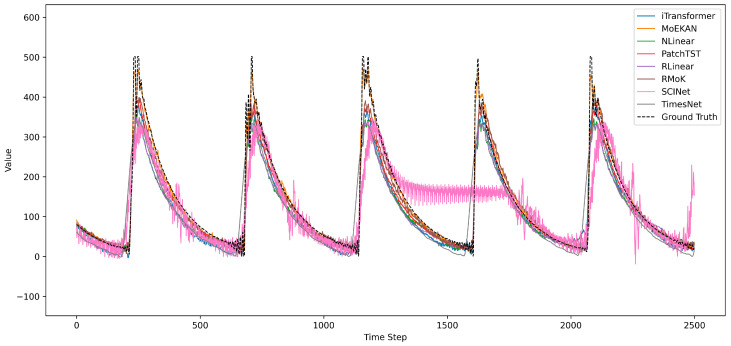
Comparison of total forecasting results between existing networks and MoEKAN.

The most notable feature is the overwhelming performance of the proposed MoEKAN model. MoEKAN achieved an MSE of 133.4579, MAE of 7.2801, and MAPE of 4.4272, which were the lowest among the errors of all the models in the comparison. In particular, the MSE of MoEKAN was reduced by over 85% and 88% relative to RMoK and PatchTST, respectively, indicating a significant decrease in the forecasting error. Its MAE and MAPE also improved to less than half of the RMoK values, with an MAPE of 4.4272%—approximately one-fifth of the ~20% MAPE of the linear models—representing an extremely low error rate. This indicates that MoEKAN achieved substantially improved forecasting accuracy compared with existing models for the OLTC vibration forecasting problem. A comparison of the total forecasting results between the existing networks and the proposed MoEKAN model is illustrated in Figure 5. The near-zero difference between the model forecasts and actual values indicates that MoEKAN effectively captured the key fluctuation patterns in the vibration signals and achieved highly accurate forecasting results. This significant performance improvement can be attributed to the innovative model architecture of MoEKAN, which integrates techniques such as RevIN, transformer-based gating, multiple KAN experts, multi-scale processing, and attention-softmax-based weighted output fusion.

**Figure 6:**
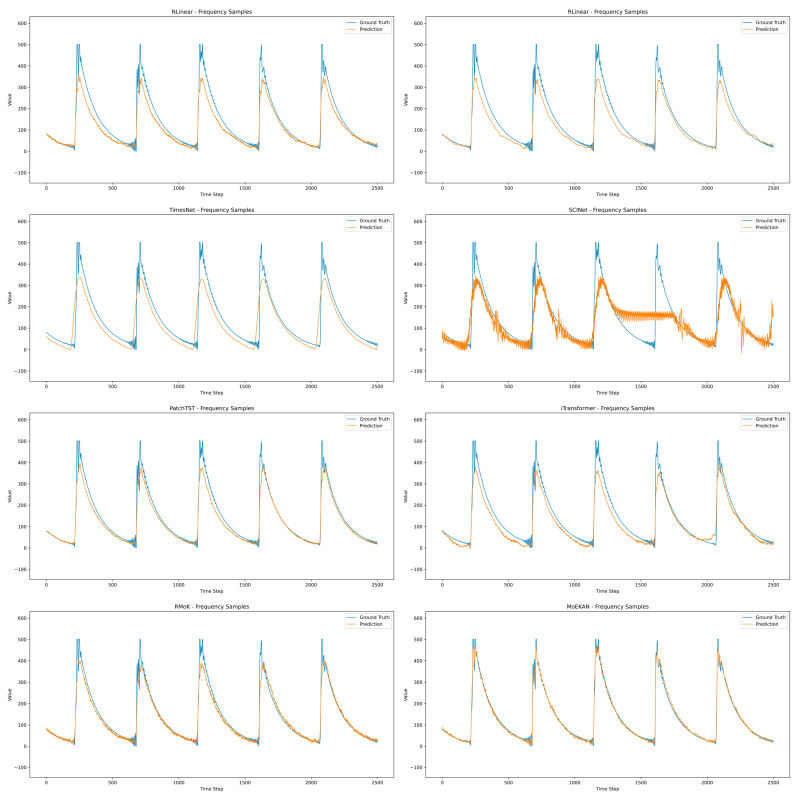
Comparison of separate forecasting results between existing networks and MoEKAN.

The outstanding performance of MoEKAN is the result of a combination of several innovative design elements. First, by incorporating RevIN to normalize the distribution of the input time series, the model mitigates the distributional differences between the training and testing data and improves the forecasting stability. By addressing nonstationarity and seasonal variations in the OLTC vibration data, RevIN enables the model to learn on a more uniform scale, enhancing its adaptability to changes in data distribution. Second, MoEKAN employs a multi-scale processing technique to comprehensively learn vibration signal patterns across different time scales. For example, by extracting features ranging from abrupt short-term vibration changes to long-term trend shifts, the model can capture both local detailed characteristics and the overall behavior of OLTC vibrations without missing any relevant information. This multi-scale feature extraction enables a more precise representation of complex vibration signals and contributes significantly to the improvement of the overall forecasting accuracy. Third, MoEKAN adopts a mixture-of-experts architecture, leveraging multiple KAN-based expert models in parallel and combining their outputs with context-dependent weighting. In particular, a transformer-based gating module is introduced to analyze the input time series in detail and dynamically determine the contribution of each expert. This gating module uses an attention-softmax mechanism to compute a weight for each expert and then produces a weighted sum of the forecasts of all experts using these weights. Owing to this dynamic gating strategy, MoEKAN can flexibly respond to diverse pattern changes in OLTC vibrations by emphasizing the forecasts of the most suitable expert or a combination of experts at each time step, yielding a level of adaptability that would be difficult for a single model to achieve. This multiexpert configuration and attention-based fusion mechanism maximizes the model’s expressiveness and serves as a key driving force behind the substantial improvement in the forecasting performance of MoEKAN. Furthermore, a comparison of separate forecasting results between the existing networks and MoEKAN is presented in Figure 6.

In summary, MoEKAN significantly outperformed all existing models in various categories across every evaluation metric, demonstrating its overall superiority in the OLTC vibration forecasting task. The integration of unique techniques—RevIN-based distribution normalization, Transformer-driven gating for specialized forecasting combinations, parallel multi-KAN expert architecture, multi-scale feature processing, and attention-softmax-based output fusion—reinforces MoEKAN’s exceptional accuracy. By incorporating these elements, MoEKAN effectively overcomes the limitations of the linear, CNN, and Transformer models, achieving both high forecasting accuracy and enhanced stability. Consequently, the proposed MoEKAN introduces a new paradigm for state forecasting in the field of vibration-based condition monitoring of transformer OLTCs, representing a significant technological advancement. The experimental results presented in this study confirm that MoEKAN architecture achieves substantially improved forecasting performance for OLTC vibration data, providing meaningful insights for the future development of deep-learning models for vibration-based diagnostics and condition forecasting of power equipment. This improvement can be attributed to the combined effect of multi-scale input processing, expert-level and scale-level Transformer gating, and the incorporation of multiple KAN basis functions (such as Taylor, Wavelet, Jacobi, and Fourier).

## 6. Conclusions

The MoEKAN model proposed in this study employs RevIN to mitigate the effects of distribution shifts in the data, dynamically adjusts the contributions of individual KAN expert modules using a transformer-based gating mechanism, and captures vibration patterns across multiple temporal scales using a multi-scale processing architecture. As a result, the proposed MoEKAN model significantly outperformed existing approaches in terms of OLTC vibration forecasting accuracy. Such enhanced accuracy provides a critical foundation for the early detection of fault indicators within the OLTC vibration data.

Despite the strong forecasting performance demonstrated in this study, the proposed MoEKAN framework has several limitations. First, the model was trained and evaluated using vibration data collected from a single OLTC configuration, and additional validation across diverse operating conditions and transformer types is needed to ensure broader generalizability. Second, the relatively low sampling frequency of the acquired vibration data limits the analysis to coarse-grained temporal patterns rather than high-frequency impulsive features that may contain additional diagnostic information. Lastly, the current work focuses on one-minute-ahead forecasting, and further investigation is required to assess the model’s robustness and scalability for longer prediction horizons or real-time field deployment.

Future work will focus on leveraging the MoEKAN-based vibration forecasting techniques to develop a real-time OLTC early fault diagnosis system that integrates both predictive and diagnostic capabilities. This system is expected to detect abnormal patterns based on the forecasting outcomes and facilitate informed maintenance decision-making, thereby enabling practical deployment in real-time operating environments. Furthermore, we anticipate that the MoEKAN-based approach is anticipated to have broad applicability, extending beyond OLTCs to comprehensive condition monitoring and early diagnosis systems for various power equipment such as transformers.

## Figures and Tables

**Table 1:** Vibration dataset information.

Train	Validation	Test
380,000	200,000	200,000

**Table 2:** Overall performance.

Type	Network	MSE	MAE	MAPE
Linear	NLinear	2127.1727	28.9779	20.2196
	RLinear	2209.7119	30.3753	21.4198
CNN	TimesNet	2939.0327	39.3729	19.9801
	SCINet	5766.9988	47.1392	23.8277
Transformer	PatchTST	1122.0353	18.1859	11.5839
	iTransformer	1542.9708	27.3423	15.8059
KAN	RMoK	907.3188	15.5203	9.3313
	MoEKAN	133.4579	7.2801	4.4272

## Data Availability

The datasets used and analyzed in the current study are available from the corresponding author upon reasonable request.
